# Interactive analysis of systems biology molecular expression data

**DOI:** 10.1186/1752-0509-2-23

**Published:** 2008-02-29

**Authors:** Mingwu Zhang, Qi Ouyang, Alan Stephenson, Michael D Kane, David E Salt, Sunil Prabhakar, John Burgner, Charles Buck, Xiang Zhang

**Affiliations:** 1 Bindley Bioscience Center, Purdue University, West Lafayette, IN 47907, USA; 2 Department of Computer Science, Purdue University, West Lafayette, IN 47907, USA; 3 Department of Computer and Information Technology, Purdue University, West Lafayette, IN 47907, USA; 4 Department of Horticulture, Purdue University, West Lafayette, IN 47907, USA; 5 Department of Chemistry, Center for Regulatory and Environmental Analytical Metabolomics, University of Louisville, Louisville, KY 40292, USA

## Abstract

**Background:**

Systems biology aims to understand biological systems on a comprehensive scale, such that the components that make up the whole are connected to one another and work through dependent interactions. Molecular correlations and comparative studies of molecular expression are crucial to establishing interdependent connections in systems biology. The existing software packages provide limited data mining capability. The user must first generate visualization data with a preferred data mining algorithm and then upload the resulting data into the visualization package for graphic visualization of molecular relations.

**Results:**

Presented is a novel interactive visual data mining application, SysNet that provides an interactive environment for the analysis of high data volume molecular expression information of most any type from biological systems. It integrates interactive graphic visualization and statistical data mining into a single package. SysNet interactively presents intermolecular correlation information with circular and heatmap layouts. It is also applicable to comparative analysis of molecular expression data, such as time course data.

**Conclusion:**

The SysNet program has been utilized to analyze elemental profile changes in response to an increasing concentration of iron (Fe) in growth media (an ionomics dataset). This study case demonstrates that the SysNet software is an effective platform for interactive analysis of molecular expression information in systems biology.

## Background

Over the past few years, biology has gone through exciting changes, rapidly moving from a "genomic" to a "post-genomic" era. Technological advances now allow collection of enormous quantities of data from all biological disciplines. These data not only provide key information about biomolecular functions, but also raise new questions concerning the relationship of these molecules. More effective use of the voluminous quantity of molecular expression data (referred to here as "'omics" data) will enable a better understanding of systems at the level of cells, tissues, organs, and organisms. A key goal in understanding (and predicting) biological behavior is represented by a relatively new discipline of systems biology that aims to provide a systems level understanding in which groups of component biomolecules and pathways are connected and operate interdependently [1].

Two essential components are featured in systems biology: powerful tools for data acquisition and computational bioinformatics. The first is represented by a large number of technologies in various fields. For example, genomics provides the list of key components (genes) available for living systems whereas transcriptomics brings information about expression levels of individual genes in certain conditions via measurement of mRNA abundance. Proteomics is the large-scale identification and characterization of gene products (proteins). Differential proteomics determines a quantitative change in abundance of proteins in a system under different conditions (e.g. diseased versus healthy) and identifies these proteins [2,3]. Metabolomics provides the identity and quantity of small molecules (metabolites) [4,5]. Ionomics provides a descriptive and quantitative elemental profile of biological systems [6]. Finally, cytomics provides the link from bio-molecules to cell function [7].

The second component of systems biology includes a growing list of data analysis and data modeling methods, leveraging the disciplines of computer science, engineering, statistics, and mathematics. For example, machine learning and text mining are significant components of computational bioinformatics that allow for connection of system elements (i.e., molecules) and modeling of networks of regulatory pathways.

Applying systems biology to biomarker discovery will increase the confidence in identified biomarkers and dramatically accelerate hypothesis generation and testing in disease models [8]. For example, this approach enables the determination, quantification and significance of biomolecules that display differences between diseased (or drug-treated) and control subjects [9]. Systems biology projects will increasingly employ parallel and comprehensive genomics, proteomics, metabolomics, ionomics, and cytomics analyses of tissue or body fluid samples. In either case, various informatics tools must be employed to collect, manage and mine experimental data [10]. A major and largely unmet goal in systems biology is to integrate results from diverse high data volume approaches (e.g., from various 'omics experiments) for correlative and comparative analyses [9].

Molecular correlation provides a powerful approach to define relationships of molecules in a biologic sample (or subject). As a simple example, two molecules will have a positive correlation if the concentration of both molecules increases in the same sample. Alternatively, two molecules will have a negative correlation if the concentration of one molecule increases while the other decreases in the same sample. Correlation of biological molecules may be linear or non-linear in nature. A common evaluation approach is to estimate molecular correlations by calculating the Pearson's correlation coefficient.

Thousands of molecules can be measured in a single 'omics experiment. Informatic tools play a critical role in extracting scientific information from the experimental data to describe molecular behaviors. Interactive visualization of molecular expression data is a critical component for 'omics data analyses. Many software packages have been developed for interactive visualization of molecular networks such as Cytoscape [11], Grapviz [12], CFinder [13], Tom Sawyer [14], VisAnt [15], and BiologicalNetworks [16]. These programs can be used to display biomolecular correlation and interaction networks. However, the existing software packages provide limited data mining capability. The user must first generate visualization data with a preferred data mining algorithm and then upload the resulting data into the visualization package for graphic visualization of molecular interactions.

Interactive visual data mining (IVDM) is a human-centered approach implemented through knowledge discovery loops coupled with human-computer interaction and visual representations [17]. It attempts to extract useful and potentially unsuspected patterns from data sets. Rather than using the data to derive certain information based on an *a priori *human knowledge structure, IVDM accommodates novel data mining goals and therefore holds great potential for systems biology. The objective of this research is to employ this approach to develop an interactive visual data mining application for 'omics expression data analyses that combines interactive visualization and statistical data mining. SysNet is the name of the system we have developed and it is able to: 1) interactively analyze intermolecular correlations using different statistical models, and 2) perform interactive comparative analysis of molecular expression data. We demonstrate application of SysNet using a simple but illustrative ionomics dataset. In this study we investigated the effects of iron concentration on the growth of *Arabidopsis thaliana *and the dependency of various elemental ion concentrations on the concentration of iron in growth medium. Pair-wise analyses of metal ion concentration and the use of SysNet revealed relevant correlation networks in this ionomics data set.

## Implementation

**Figure 1:**
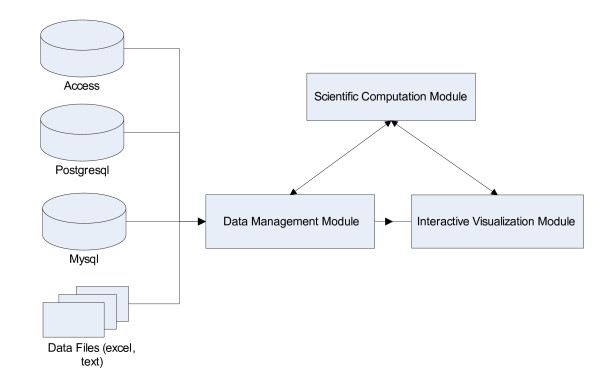
Architecture of SysNet software.

SysNet was developed in Microsoft Visual Studio .Net using Visual C++ to allow fast generation of forms. Figure 1 shows the architecture of SysNet software. The inputs for SysNet can be data stored in various databases including Access, Postgresql and MySQL, or data files, such as text or Excel files. There are three modules in SysNet: data management, scientific computation, and interactive visualization (Fig. 1). The data management module connects data from the various databases and files. It also communicates with the scientific computation module to obtain the intermediate computational results. The scientific computation module comprises a library of scientific computation algorithms. For example, computation of correlation and data model fitting is done by the scientific computation module. The interactive visualization module is the core of the system; it takes information from the data management and scientific computation modules and provides dynamic visualization on the computer screen.

The input data contains four data tables for expression, molecular, sample and experimental information, respectively. Molecular expression information generated from different 'omics experiments such as proteomics, metabolomics and ionomics, is concatenated into a single table, which contains all normalized expression data for each molecule, such as aligned peak tables in case of proteomics or metabolomics [18]. The molecular information table contains descriptive information about each molecule. In the case of a protein, this will include accession number, name(s), amino acid sequence, etc. The sample table contains all meta information of each sample. For instance, it may include patient clinical information, sample origination site, etc. The experiment table contains all key analytical and experimental parameters.

There are two major functionalities in the current version of SysNet: interactive analysis of molecular correlations and comparative analysis of 'omics expression data. These functions were developed as two distinct forms that share multiple analysis and visualization routines. For both functionalities, SysNet enables the user to interactively select the interested molecules from the graphic display window. All related information of the selected molecules is automatically updated in the graphic display. SysNet can export all graphic presentations as jpeg, bitmap, or gif images. It also exports the molecular correlation values as a matrix in text format.

For correlation analysis, SysNet automatically calculates pairwise correlation coefficients for all possible molecular pairs using one or more available correlation methods with the uploaded data. The calculated correlation coefficients are stored in computer random access memory (RAM) for easy access during interactive visual analysis. For comparative analyses, SysNet automatically groups expression data based on a user-assigned experimental identification number (EIN), which is recorded in both the expression and experiment information data tables. The EIN is a designation applied to all identified molecules detected in particular comparative experiments. All expression data with the same EIN are further categorized based on biological data type, e.g., proteomics, metabolomics and ionomics data. Each data type can be further sub-categorized if necessary.

The location of graphical entities such as data points or icons in a display can convey significant information about the relations between entities. Entity placement is therefore a critical consideration for data visualization. Many display techniques are available including hierarchical, symmetric, orthogonal, circular layout and others. Hierarchical ordering relations can be explicit, as in organizational charts or directory structures; or derived, as for example from clustering or partitioning algorithms. However, hierarchical ordering requires that a leaf graphical entity should not have a direct relationship with other than parent graphic entities. Thus a leaf graphical entity can only have indirect relationships with other entities through its parent graphical entities. Compared with hierarchical layout, the circular layout enables each graphical entity to have a direct relation with any other graphical entity as well as indirect relationships with other graphical entities through its parent entities. This feature of the circular layout makes it an ideal choice for molecular correlation networks, where molecules may correlate more or less strongly with many other molecules.

Spring embedding [19] is another popular layout algorithm which can be used to display molecular correlation networks. The drawing process considers the graph as a force model system which includes repulsive and attractive forces. The effect of spring embedding is to distribute nodes in a two-dimensional plane with some separation, while attempting to keep connected nodes reasonably close together. The advantage of this algorithm is that it is easy to see molecular correlation clusters, such as groups of molecules that are connected to each other. A disadvantage of this approach is the molecules detected in different experimental groups will be mixed and displayed on the screen based on the force system. The user must therefore navigate through the entire correlation network to find the interesting molecules.

Although symmetric and orthogonal layouts may also effectively display molecular correlation networks, the current version of SysNet visualizes 'omics expression data as a two-dimensional network [20] supporting a circular layout, where molecular species are represented as nodes located on circles. Intermolecular correlations are represented as links or edges between nodes. The circular layout is also advantageous for visualization that centers on large numbers of molecules. However, large numbers of edges connecting vertices on a circle inherently results in overlaps. For this reason, SysNet provides a heatmap layout as an alternative graphic visualization method.

## Experimental Methods

*Arabidposis thaliana *plants were seeded (N = 12) into 20-row plastic trays, stratified for 3 days at 4°C and allowed to grow for 5 weeks at 19 to 22°C under 90 μEm-2s-1 of fluorescent light. The growth medium was Sunshine Mix LB2 (Carl Breholb & Son, Indianapolis, IN) which had been spiked with As, Cd, Li, Ni, Pb and Se. Plants were watered twice per week with quarter-strength type 2 Hoaglands where the normal iron was replaced with 0.5 to 30 μM Fe-HBED (N, N'-Di(2-hydroxybenzyl) ethylenediamine-N, N'-diacetic acid monohydrochloride hydrate (Strem Chemical, Inc.) mixed with an equimolar amount of iron (III) nitrate (Alfa Aesar) and brought to pH 6.0 with 4 M KOH.

Three mg (dry) of each plant were transferred into Pyrex tubes (16 × 100 mm) and dried at 92°C for 20 hr. After cooling, 7 of 108 samples from each tray were weighed. All the samples were digested with 0.7 ml of nitric acid (OmniTrace, VWR) and diluted to 6.0 ml. Elemental analysis was performed with an inductively coupled plasma – mass spectrometer (ICP-MS) (Elan DRCe, PerkinElmer) for Li, B, Na, Mg, P, K, Ca, Mn, Fe, Co, Ni, Cu, Zn, As, Se, Mo, and Cd. Ten samples from each run were retained and run as a unit at the end of the experiment to facilitate cross-tray comparisons. All samples were normalized to calculated weights, as determined with an interactive algorithm using the best-measured elements, weights of the 7 weighed samples and the solution concentrations.

## Results

SysNet provides interactive analysis and graphic visualization of molecular expression data. There are two major functionalities in the current version of this software: interactive analysis of molecular correlations and comparative analysis of 'omics expression data.

*Interactive analysis of molecular correlation *– Measures of molecular correlation are descriptive statistics that represent the degree of relationship between two or more variables, but are not inferential statistical tests. Parametric and nonparametric statistical methods are available for correlation measurement [21]. The parametric method is based on assumptions that include 1) the subjects are randomly selected from the population; 2) the size of subjects is large enough to represent the distribution of a population; and 3) the variables have a bivariate normal distribution. Nonparametric or parameter-free methods do not rely on the estimation of parameters (such as the mean or the standard deviation) but describe the distribution of the variable of interest in the population.

SysNet has implemented both parametric and non-parametric pairwise measures including the parametric Pearson product-moment correlation (*r*_*p*_), the non-parametric Spearman correlation (*r*_*s*_) and the non-parametric Kendall's coefficient of rank correlation (*τ*).

Pearson product-moment correlation coefficient (*r*_*p*_) assumes that a linear function best describes the relationship between two variables. It can be used to evaluate data for *n *subjects, each of which has contributed a score on two variables designated as *X *and *Y*. *r*_*p *_is calculated as follows:

$$r_{p}=\frac{\sum XY-\frac{\left(\sum X\right)\left(\sum Y\right)}{n}}{\sqrt{\left[\sum X^{2}-\frac{{\left(\sum X\right)}^{2}}{n}\right]\left[\sum Y^{2}-\frac{{\left(\sum Y\right)}^{2}}{n}\right]}}$$

Spearman's rank-order correlation (*r*_*s*_) is a bivariate measure of correlation employed with rank-order data. It determines the degree to which a monotonic relationship exists between two variables. Equation (2) shows the *r*_*s *_calculation for *n *subjects where each subject has an *X *and a *Y *score. The rank of *n *subjects scores on the *X *and *Y *variables are recorded in *R*_*X *_and *R*_*Y*_, respectively. $d=\left\{R_{X_{i}}-R_{Y_{i}},i=1\cdots n\right\}$ contains a difference score for each subject that is obtained by subtracting a subject's rank on the *Y *variable from the subject's rank on the *X *variable.

$$r_{s}=1-\frac{6\sum d^{2}}{n\left(n^{2}-1\right)}$$

The non-parametric Kendall's coefficient of rank correlation (*τ*) is also a bivariate measure of correlation employed with rank-order data. In this case, one assumes that data are in the form of the following two pairs of observations expressed in a rank-order format: a) $\left(R_{X_{i}},R_{Y_{i}}\right)$ (that represents the ranks on variables *X *and *Y *for the *i-*th subject, respectively); and b) $\left(R_{X_{j}},R_{Y_{j}}\right)$ (that represents the ranks on variable *X *and *Y *for the *j*-th subject, respectively). If the sign of the difference $\left(R_{X_{i}}-R_{X_{j}}\right)$ is the same as the sign of the difference $\left(R_{Y_{i}}-R_{Y_{j}}\right)$, a pair of ranks is said to be concordant. If the sign of the difference $\left(R_{X_{i}}-R_{X_{j}}\right)$ is not the same as the sign of the difference $\left(R_{Y_{i}}-R_{Y_{j}}\right)$, a pair of ranks is said to be discordant. *τ *is calculated as follows.

$$\tau =\frac{n_{C}-n_{D}}{\left[\frac{n\left(n-1\right)}{2}\right]}$$

where *n*_*C *_is the number of concordant pairs of ranks. *n*_*D *_is the number of discordant pairs of ranks, [*n(n-1)/2*] is the total number of possible pairs of ranks.

The Kendall coefficient *τ *is equivalent to Spearman's *r*_*s *_with regard to the underlying assumptions and the two are comparable in terms of statistical power. However, Spearman *r*_*s *_and Kendall *τ *are usually not identical in magnitude because the underlying logic and computational formulas are different. Importantly, Kendall *τ *and Spearman *r*_*s *_may lead to different interpretations. Spearman *r*_*s *_can be thought of as the regular Pearson product moment correlation coefficient in terms of proportion of variability accounted for, except that Spearman *r*_*s *_is computed from ranks. Kendall *τ*, on the other hand, represents the difference between the probabilities that in the observed data two variables are in the same order versus different orders.

In an 'omics global profiling experiment, multiple samples (subjects) will be analyzed and many molecules (observations) detected in each sample. These molecules can be proteins, metabolites and/or metal ions, etc., depending on experimental design. Even though the experimental analyses vary significantly in different types of omics research, the final expression data are similar. Basically, multiple molecules will be detected in each sample and each detected molecule has a digital value indicating the relative expression level of that molecule in the sample. The molecular expression data are then organized as a data table. For example, the column represents samples while each row stores the expression values of a specific detected molecule in each sample. We selected a relatively simple tabular ionomics dataset to illustrate the capability of SysNet. The software can be applied for visualization and correlation of data from all such high volume molecular expression experiments, including proteomics and metabolomics.

**Figure 2:**
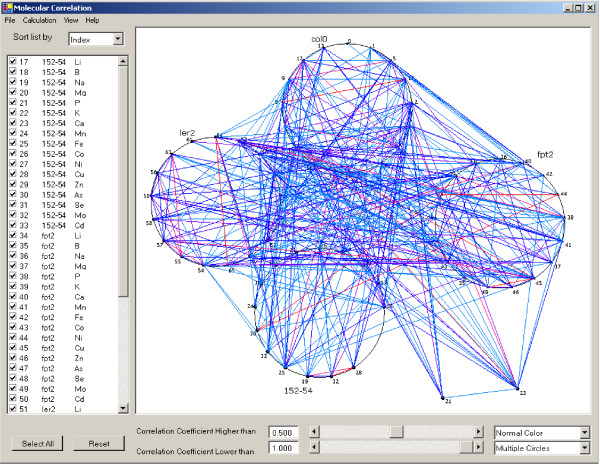
Interactive display of molecular correlation in multiple circles with the normal SysNet color schema. The left panel displays molecular information and the right panel displays molecular correlations. Each circle represents elements measured in one *Arabadopsis *strain (i.e., in each experimental group). Col0 is a wild-type plant whereas Ler2, fpt2 and 152–54 are mutants.

For interactive analysis of intermolecular correlations, SysNet focuses on expression data from a single experiment, where multiple subjects are used for analysis. By default, SysNet calculates Pearson product-moment correlation (*r*_*p*_) between every two molecular pairs. The user is able to select other methods based on the nature of data. These measurements are computed dynamically and stored in RAM. The molecular correlation is displayed in a 'main window' that is divided into two panels (Fig. 2). The left panel lists all molecules measured while the right panel displays the molecular correlation. The circumference of each circle is proportional to the number of molecules to be displayed.

Molecular correlation analysis evaluates the concentration change of different molecules in all samples. The maximum number of pairwise correlations among these molecules can be represented as *n(n-1)/2*. In our ionomics experimental setup 17 elements are measured for each sample. Figure 2 displays correlation networks for four *Arabidopsis *strain experimental groups: ler2, col0, 152–54 and fpt2 with just 68 elements displayed. This visualization will become extremely busy if thousands of correlations are displayed. For this reason, we implemented three methods for visual analysis of large numbers of correlations: one is to filter correlations based on correlation strength, the second is to create a larger image using zooming functions, the third enables the user to move a molecule (node) or an experiment category (circle) around to facilitate visualization. The two sliding bars at the bottom of the screen determine the correlation coefficient value used to filter the data displayed. All molecules having at least one correlation coefficient higher than the filtering criteria will be displayed as a node. The user can adjust the filter values either by moving the sliding bar or by entering a number at the bottom of the right panel. Molecular and correlation information is automatically updated on the graph in response to user changes. In the second approach, SysNet changes the size of the correlation map with zooming functions that enable the user to perform focused analyses. The user can also re-arrange the correlation map by simply selecting a circle or node and dragging it to another panel location. SysNet displays all nodes on a circle by default. Figure 2 is a screen shot showing that node 21 and 23 have been moved from their default location on the circle to another screen location for easy visualization.

Molecular profiling 'omics experiments include very many molecules, only some of which are of interest to biologists. For this reason, SysNet enables the user to add or remove a molecule by changing the status of the check box in the left panel. If a molecule is unchecked in the left panel, the node in the right panel representing that molecule and all correlation edges related with that molecule will disappear and the entire correlation network will be re-arranged. If an un-checked molecule on the left panel is checked, that molecule will be randomly inserted into the corresponding graphic display and the entire correlation network updated.

**Figure 3:**
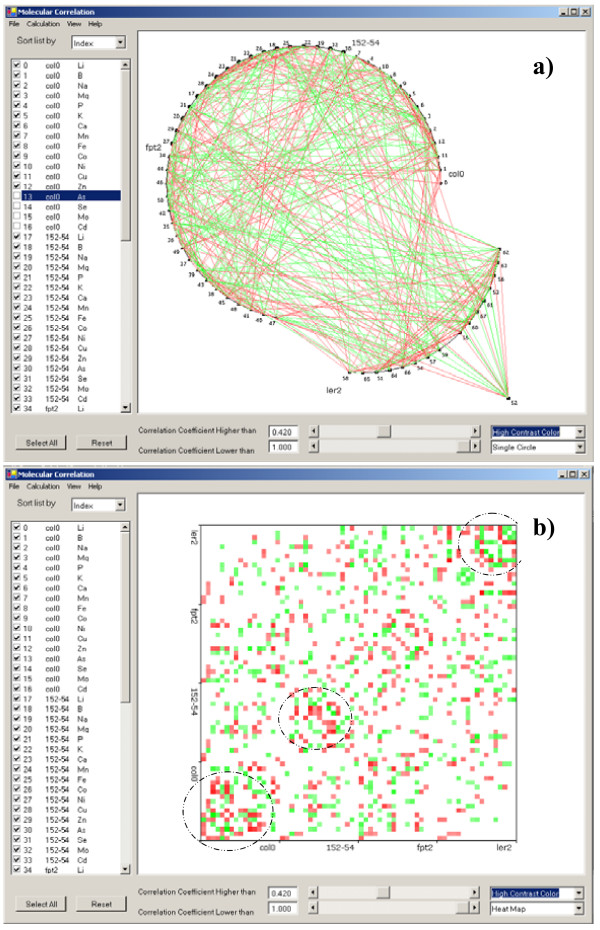
Graphic display of the same molecular correlations in (a) one circle and (b) heat map with high contrast color schema. High contrast color schema is used to show the correlation directions. Green indicates positive correlation while red indicates negative correlation. The encircled areas in panel (b) readily demonstrate clusters of strong molecular correlation.

Three models are available for display of the correlation map in the main window: multiple circles, single circle, and heatmap. The multiple circle display enables effective usage of screen space (Fig. 2). Each circle of the multiple-circle display shows all molecules belonging to a single experimental group (the *Arabadopsis *strains col0, ler2, fpt2 and 152–54, in this case). In the single circle display (Fig. 3a), all molecules with the same EIN recorded in the input database or data files are displayed in one circle, with breaks in the circle representing divisions between the different experimental groups. All molecules from the same experimental group are displayed in the same arc. Each arc and molecular node can be re-arranged to ease visualization.

The disadvantage of circular display is the overlap of molecular indexes (software-assigned numbers to represent molecules in a graphic display) that may obscure visualization of correlations with these molecules. It is easier to see correlation patterns in a heat map display when dealing with large numbers of molecules. For example, three intense color regions are apparent along the diagonal indicating elements that are strongly correlated within experimental-sets (Fig. 3b; highlighted with dotted circles). It is not easy to recognize this pattern in the circular display (Fig. 2 and Fig. 3a). The disadvantage of the heat map is that all molecules are displayed on one axis so that it is difficult to see details of correlations for a single molecule if a large number of molecules are included. This problem is overcome in SysNet by creation of a large correlation map using the zooming functions.

Two color schemas are implemented to visualize the correlation strength: normal (Fig. 2) and high contrast (Fig. 3). The normal color scheme focuses only on the absolute value of correlation strength with white indicating zero and red indicating a correlation strength of 1. The high contrast color scheme differentiates positive (green lines) and negative correlations (red lines).

**Figure 4:**
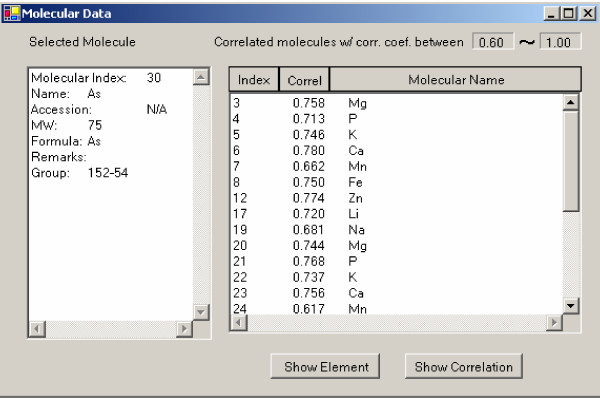
List of molecules that correlate with the molecule of interest. The left panel shows information for the molecule of interest. All correlated molecules are displayed in the right panel. By highlighting a molecule in the right panel, the user can either invoke another molecular window of the highlighted molecule by clicking the 'Show Element' button or invoke a correlation window by clicking the 'Show Correlation' button.

To investigate the details of a specific molecule of interest, SysNet provides two visualization methods. By clicking a node (i.e., molecule of interest) in either circular or heat map layout on the graph in the main window, a molecular window will pop up with a list of details for that molecule in the left panel and information about correlated molecules in the right panel (Fig. 4). The filtering criteria for molecular correlation coefficients in this window are the same as specified in the main window and are indicated in the upper right of the screen. Multiple sorting functions are provided for the correlated molecules (right panel) including sorting by molecular index, correlation values (Correl) and molecule name. With a double-click on the selected molecule, SysNet brings up a web browser displaying the search results for the corresponding molecules from public databases relevant for the type of molecule. For example, the current version of SysNet displays protein information from UniProt database [22], metabolite information from KEGG [23], and gene information from GenBank [24]. The user can highlight a molecule displayed in the right panel with a single click. A molecular information window for the highlighted molecule can then be evoked by clicking the 'Show Element' button. Correlations for that molecule are displayed in another window upon clicking of the 'Show Correlation' button.

**Figure 5:**
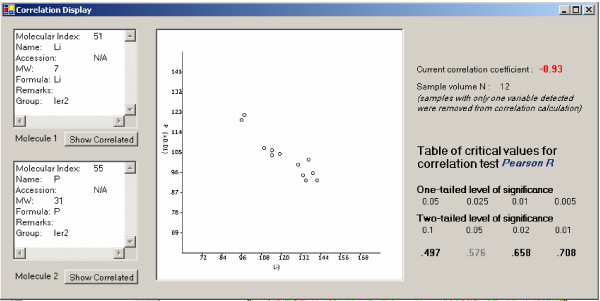
Correlation between two elements (Li and P) in the ler2 *Arabadopsis *strain. The information for the two correlating elements is displayed in the list boxes on the left. There are 12 ler2 ionomics samples. The middle graphic shows the expression data of the two elements, with the level of Li on the x-axis and P on the y-axis. Each point therefore represents the expression levels of Li *and *P in the same sample. Statistical correlation information is listed on the right side of the screen. The critical values for the correlation test are also displayed to facilitate assessment of the statistical significance of the selected correlation.

SysNet also allows the user to view details of a correlation by clicking on a correlation edge on the graph in the main window to invoke a correlation window, which displays details of the two correlated molecules and a graph showing molecular expression levels for the two molecules measured in different samples. Figure 5 shows a correlation between the elements Li and P in the ler2 strain. Elemental information of these two elements is displayed in the two list boxes on the left. There are 12 ionomic samples from the *Arabidopsis *strain ler2. Each dot in the middle graphic display represents the expression level of the Li (x-axis) and P (y-axis) in one sample. Apparent negative correlation of these elements in this strain is indicated in the graphic; as P levels increase, Li levels decrease and vice versa. The table of critical values for a selected statistical test is automatically displayed on the right side of the screen to enable the user to evaluate the significance of the current correlation. The molecular window and the correlation window may also call each other with the "Show Correlated" button enabling the user to toggle between these information resources.

*Comparative analysis of omics expression data *– SysNet also enables researchers to interrogate comparative molecular expression studies. This may include any study that monitors molecular behavior under different conditions: platform comparisons, treatments, drug effects, time lapse, etc. Multiple samples are typically analyzed in parallel for 'omics studies, as is the case with our ionomics study. This experimental design enables scientists to understand both the technical and inter-sample variation. For SysNet comparative analyses, all expression data to be compared are concatenated into a single expression data table, where EIN is used to differentiate data for comparison.

**Figure 6:**
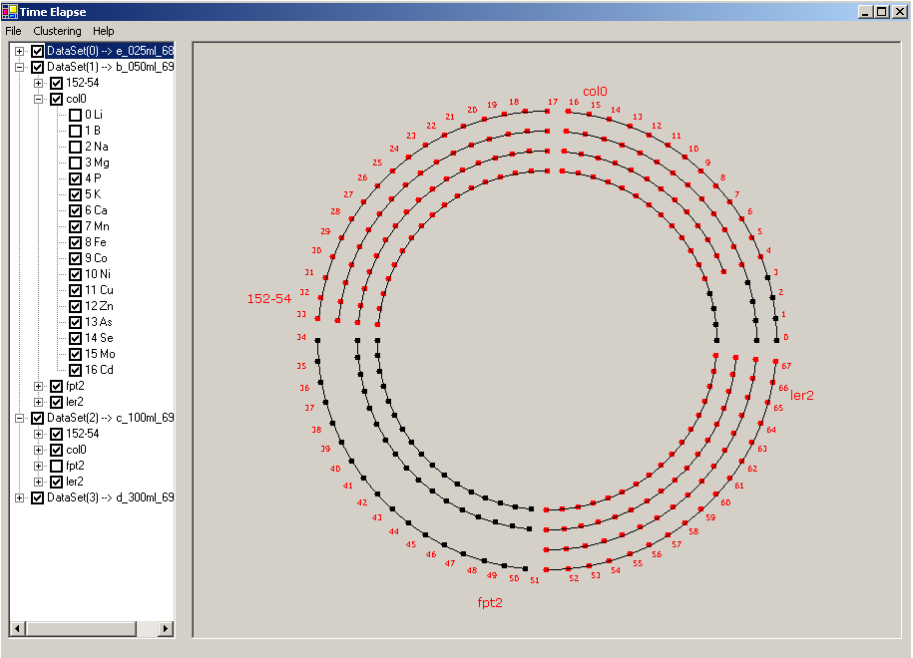
Comparative analysis of 'omics expression data. Molecules are aligned in concentric circles based on molecular name and experimental group. Experimental groups (sets) are displayed as concentric arcs of the circles with each circle representing a separate comparative experiment. Molecules indicated in red are identified in each experiment in the set. Molecules indicated in black were not identified in all comparative experiments. The outer most circle provides an index of every molecule detected in the comparative studies with a designated index number which can be used to find the relevant experimental and molecular information on the left panel lists. The user can interactively select or de-select molecules and experimental groups in the left panel, and the related information will be automatically displayed in the graphic.

SysNet aligns molecules based on molecular name and experimental groups. The aligned molecules are displayed in multiple concentric circles, where each circle includes all molecules measured in the same comparative experiment, i.e., having the same EIN. Each circle in the graphic is separated into multiple segments representing the different experimental groups (Fig. 6). The experimental information panel is displayed with a tree structure on the left side of screen. The root of this structure is each EIN composed of information from multiple experimental groups such as col0, ler2, fpt2 and 152–54. Each experimental group contains molecular information of each molecule analyzed in the experiment, e.g. molecular index and molecular name. The molecular information is static, but the user can change the check status of an EIN or experimental group to decide whether the related molecular information should be displayed in the graphic panel.

Red coloring in figure 6 is used to indicate molecules detected in every experimental group in the comparative experiments while black indicates a molecule that was not detected in all experimental groups. If a molecule is not detected in any experimental group, or the molecule is deselected in the experimental information panel, that node does not appear in the graphic display. An index number of all molecules detected in a comparative experiment is displayed in the outermost circle. The designated index number may be employed to find molecular and experimental information in the experimental information panel.

Displaying all molecules in multiple concentric circles enables experimental information for each molecule to be easily categorized by location on the circle. This design also enables the user to perform interactive visual data analysis by simply clicking on the node representing each molecule of interest. However, the concentric circle display will become congested with large numbers of molecules. To address this problem, the SysNet zoom function may be employed to display the concentric circles in a larger graph. The zoom function is invoked by a single mouse right click.

**Figure 7:**
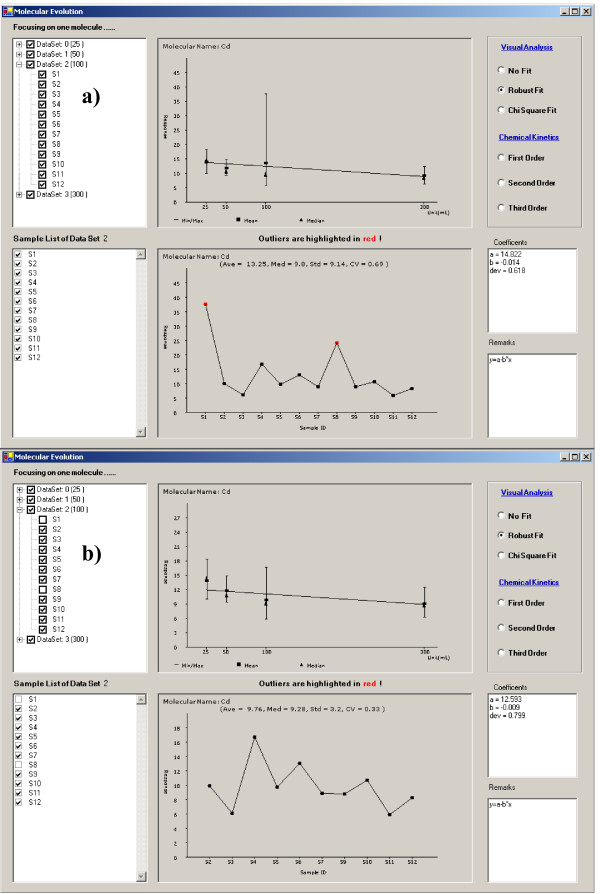
Display of molecule behavior in a comparative analysis. The second comparative experiment labeled as DataSet 2 on the upper left panel is the active experiment group including the molecular expression levels of the molecule of interest (cadmium, Cd) in each sample displayed in the lower graphic panel. a) the expression levels of this element in samples 1 and 8 were detected as outliers and highlighted in red; b) the outliers in the active experiment have been removed by un-checking S1 and S8 in the left panel.

The user can focus on the behavior of a single molecule in multiple experiments. By clicking a node on the graph of the comparative window (Fig. 6), a multiple panel 'Molecular Evolution' window will appear that displays the expression information for that molecule in each experiment (Fig. 7a). The upper left 'dataset panel' displays EIN, experimental groups, and individual experimental samples. In the upper graphic is shown the behavior of the molecule of interest in multiple samples including response range, average and median expression level value in each comparative experiment displayed. The user may add or remove molecules using check boxes in the dataset panel. If an EIN is unchecked, expression level information for all samples in that comparative experiment will be assigned as zero in the graphic display. This information is also reflected in the lower left 'sample list' panel that displays all samples being analyzed in the an experimental group, which is highlighted in the dataset panel by a single-click on the experimental group. The molecular expression level in each sample is displayed in the lower graphic. In this graphic, the user may remove the molecular response detected in a sample by unchecking the specific sample box in the sample list panel. This information is automatically updated in the two graphics on this screen.

Molecular expression level detected in analytical instruments may be affected by many factors during data acquisition and analysis. We used Sprent's equation [21] to find statistical outliers in sample replicate experiments:

$$\frac{\left|X_{i}-M\right|}{MAD}>Max$$

where *X*_*i *_is molecular expression data being evaluated as a potential outlier, and *M *is the median of the molecular expression data in all samples. *MAD *is the median absolute deviation, and *Max *is the threshold value that must be exceeded to conclude that the value *X*_*i *_is an outlier. The value *Max *is set as 50, which is extremely likely to identify molecular expression data that deviates from the mean by more than three standard deviations.

Molecular expression data points identified as outliers are highlighted in red in the Molecular Evolution screen lower graphic (Fig. 7a). The user can remove outliers by un-checking the corresponding sample names in the Sample List panel. Figure 7b displays molecular behavior after the samples containing outlier molecular expression data have been removed (S1 and S8). Manually removing samples containing outlier molecular expression data can be an inefficient method of data selection when dealing with a large number of molecules. Therefore, SysNet automatically removes all samples containing outlier molecular expression data and the check box of each sample containing the outliers on the left panel is un-checked. The user can re-visit these outliers by checking the corresponding sample box. The graph in the upper central portion of Figure 7a displays the molecular concentration evolution for a time course study. In our example, this graph displays the concentration dependency of the element Cd, with the concentration of Fe in growth medium.

SysNet also provides quantitative modeling to evaluate the profile of molecular responses. We have implemented algorithms to model chemical kinetics for first order, second order and third order chemical reactions evaluated on a molecule-by-molecule basis. Chemical kinetics describes how the rate of a reaction varies with the concentrations of various reactants in the system. The rate of reaction is proportional to the rates of change in concentrations of the reactants and products; that is, the rate is proportional to a derivative of a concentration. This approach can be used to model simple biological process. More sophisticated models will be implemented in future.

The implemented visual analysis approaches are non-quantitative and used in cases where the molecular concentration profile can not be modeled based on accurate and absolute quantification. In our study, we investigate the metal ion concentration change in growth medium with different Fe concentrations. There are many biological processes involved in establishing the final concentration of each metal ion and in many cases, quantification of molecular expression levels for each of these biological processes is not available. The visual analysis approach however, enables us to identify the trends of metal element absorption with the increase of Fe concentration in growth medium. SysNet implements three functions for visual analysis: not fitting, robust fitting and chi square fitting (Fig. 7) [25]. Both robust- and chi square- fit the molecular response to a straight line. Analysis of all elements in each group indicated that the concentration of Fe in the growth medium differentially effects elemental profiles in the col0, fpt2, 152–54 and ler2 experimental groups. For example, with increasing Fe concentration in the growth medium, the concentrations of Cd, Co and As in mutant 152–54 decrease. This suggests that the elemental ion absorption pathways of Cd, Co and As are related with the growth medium in 152–54 mutant. The concentration of other elements did not show a significant dependency on the concentration of Fe in the growth medium. It is interesting that the concentration of Fe in the plant does not vary significantly with the increase of the concentration Fe in the growth medium. This indicates that the process of absorption of elemental ions is selective. Details of these experimental analyses related to the mechanisms of elemental ion absorption will be reported separately. We have also employed SysNet to study protein and metabolite correlation networks in proteomics and metabolomics data sets.

The current version of SysNet is developed in Microsoft Visual Studio .Net using Visual C++. Most data file types and database sources can be employed as its input. The system is therefore open for analyses by the vast majority of users. To further expand the application of SysNet, we plan to develop a Unix version of SysNet using Java.

## Conclusion

SysNet takes data from high volume molecular expression experiments as its input and enables interactive visual data mining of molecular correlations. Correlations are presented with circular and heatmap layouts. The software provides a common framework, allowing presentation of molecular correlations from multiple 'omics experiments in a single environment. The user is free to restrict the viewed items based on correlation strength, and further by simply deselecting specific items. SysNet also provides capability for comparative analysis of molecular expression data that can be applied to platform comparison, drug effects, life cycle studies and more. SysNet is able to export all of its graphic presentations as images and exports molecular correlation information as a matrix in text format. As a data mining tool for molecular expression studies, SysNet has been successfully used to indicate and investigate elemental level correlations in plant samples and the dependency of elemental levels on the concentration of iron in growth medium. Although there is a significant concentration dependency between some elemental ions and iron in the growth medium, the concentration of iron in the plant did not vary significantly under these conditions. This indicates the selectivity of the process(es) of absorption of elemental ions in plant tissue.

## Availability and requirements

Project name: SysNet

Project home page: 

Operating system: Window XP

Programming language: Microsoft Visual .Net C++

License: SysNet is free to academic research.

Restrictions to use by non-academics: Permission from the corresponding author is needed.

## Authors' contributions

XZ designed the software and project. MZ, QO, and AS implemented the software and participated in the data analysis. DS performed laboratory experiment to analyze metal elemental profile. MK, SP, JB, CB, and XZ prepared the manuscript. All authors read and approved the final manuscript.
